# Self-Motion Versus Environmental-Motion Perception Following Rotational Vestibular Stimulation and Factors Modifying Them

**DOI:** 10.3389/fneur.2019.00162

**Published:** 2019-02-27

**Authors:** Ognyan I. Kolev

**Affiliations:** ^1^University Hospital of Neurology and Psychiatry, Sofia, Bulgaria; ^2^Institute of Neurobiology, Bulgarian Academy of Sciences, Sofia, Bulgaria

**Keywords:** self-motion, environmental-motion, perception, insinuation, vestibular, visual

## Abstract

Motion perception following rotational vestibular stimulation is described either as a self-motion or as an environmental-motion. The purpose of the present study was to establish frequency of occurrence of both sensations in healthy humans; what other sensations they experience and how factors insinuation and visual cues modify them. Twenty-four healthy subjects were rotated with constant velocity of 80°/s in four combinations of opened and closed eyes during the rotation and after a sudden stop. After the cessation of the rotation they reported their spontaneous or insinuated illusory motion. During spontaneous perception after sudden cessation of rotation and with the subject's eyes open, the illusory sensations of self- and environmental-motion were almost equally presented. There was no simultaneous illusory perception of self-motion and environmental-motion. Insinuation modified the perception of motion; presence or absence of visual cues prior to the cessation of the rotation and the presence or absence of visual cues immediately after the cessation of the rotation changed the motion sensation. There is a gender effect in motion perception. This finding might be of benefit in further exploring the gender difference in the susceptibility to motion sickness.

## Introduction

The vertigo in which a subject inappropriately experiences the perception of motion is generally due to a dysfunction of the vestibular system or its unusual stimulation. The vertigo is used to describe two different types of motion: “external” vertigo—false sensation that the visual surround is moving, and “internal” vertigo—false sensation of self-motion (1, 2).

Vertigo, with its two types, is unique due to the fact that, unlike other pathological symptoms, it may be experienced by healthy humans during strong stimulation of the vestibular system in their life activities, e.g., repetitive spinning, known as a physiologic vertigo (3). Physiologic vertigo occurs with the physiological stimulation of any of the three stabilizing sensory systems: vestibular, visual or somatosensory. It is induced by intersensory or intrasensory mismatches (4). Unlike in most cases of vestibular pathology, in healthy humans it is caused by symmetrical stimulation of both labyrinths. In one, generating excitatory impulses, in the other—inhibitory impulses.

Numerous articles describe the characteristics of vertigo in pathology (5–7). This sensation belongs to so called derealization symptoms, which is discussed in previous articles not only in vestibular pathology but also in psychiatric practice, for instance anxiety [see in (8)]. However, to understand the pathology better, as well as to understand the physiological vertigo, importantly linked to the development of devices for enjoyment and human transportation—especially aircrafts and spacecrafts where spatial disorientation caused by motion illusions may lead to accidents (9, 10), we need to know how this sensation is experienced by healthy humans.

The search in the literature shows that there are very few publications in this field (11–20) which are mostly focused on self-motion perception. An earlier study on illusory self-motion perception shows that it significantly varies when strong unilateral caloric vestibular stimulation is applied in healthy humans (12, 21). However in the study the illusory environmental-motion perception was not investigated. Besides the subjects were either in supine position or their head was tilted backward so that the lateral semicircular canals were in the vertical plane therefore their afferentation interferes with the signal from the otoliths due to the effect of the gravity.

In a previous investigation (11) it has been shown that at threshold level of vestibular or visual stimulation the insinuation changes motion perception in healthy humans. Therefore it is of interest to know whether and in which way the insinuation during supra-threshold stimulation will change the perception.

This study aimed to establish which sensation between self- and environmental-motion predominates in healthy humans when the body is suddenly stopped after vertical axis rotation, what other sensation is experienced, the direction of motion with respect to the stimulus direction, and how the perception is influenced by different conditions of vestibular-visual interaction. We also aimed to establish whether insinuation influences the perceived motion and is there a gender effect. We hypothesize that perception for motion in healthy humans will not be equal for all humans but supposedly exists dominating perception of what is moving and its direction, indicating inter-individual difference; the visual-vestibular interaction and insinuation will change specifically the perception; a gender effect exists mainly in insinuation.

## Subjects and Method

Twenty-four healthy volunteers (12 men and 12 women with the same age range: from 23 to 35 years) took part in the present study. Their vestibular and visual systems were examined prior to the study in order to exclude any disease affecting these systems. The subjects in seating position were rotated on a Barany chair surrounded with a stationary optokinetic pattern with vertical black and white stripes in an illuminated room. The rotation was 12 cycles with a constant velocity of 80°/s−54 s. totally, twice to the left and to the right randomized between the subjects. After a sudden cessation of the rotation they had to describe their sensation of motion.

Twelve experimental series randomized between the subjects were conducted. Three randomized questions were asked: (1) What is moving and in what direction? (spontaneous perception); (2) Are you moving and in what direction? (insinuated perception); (3) Is the environment moving and in what direction? (insinuated perception). In the insinuated series the subjects were instructed to attend to, and to report the occurrence of either self-motion (self-motion task) or environmental-motion (environmental-motion task). We aimed to investigate, with the last two tasks, the effect of the insinuation factor (11). The tasks were performed under four conditions with respect to the visual input factor, randomized between the subjects, in order to understand the effect of the integration of the vestibular signal with the visual one. These four conditions can be summarized as follows:

1. Eyes open during rotation and after the sudden stop
2. Eyes closed during rotation then eyes open after the sudden stop
3. Eyes open during rotation then eyes closed after the sudden stop
4. Eyes closed during rotation and after the sudden stop.

When the subjects had their eyes open they were instructed to look straight ahead. For the purpose of the analysis, the reports were divided into four possible groups with respect to the presence and the direction of motion. The groups were as follows: (1) rotation in the opposite direction to the chair rotation, (2) rotation in the same direction as the chair, (3) perception of some motion without clear discrimination of the direction of the motion or what is moving, (4) lack of perception of motion. The gender effect was also investigated. The *Chi-square test* was used for statistical analysis. A *p* < 0.05 was accepted as significant.

The study was approved by the local Bioethics Committee of the Institute of Neurobiology of The Bulgarian Academy of Sciences, Sofia. It was performed in accordance with the ethical standards laid down in the 1964 Declaration of Helsinki. Written informed consent was obtained from all subjects of this study.

## Results

**Figure 1:**
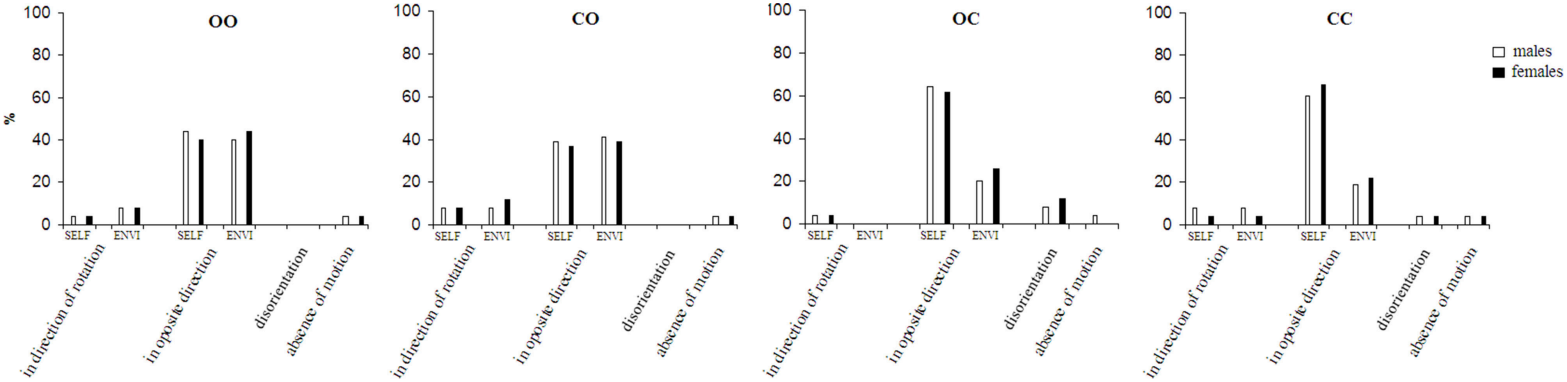
Spontaneous perception of motion after sudden stop of rotation. OO, eyes opened during the rotation and after the sudden stop; CO, eyes closed during the rotation and opened immediately after the sudden stop; OC, eyes opened during the rotation and closed immediately after the sudden stop; CC, eyes closed during the rotation and after the sudden stop; SELF, perception of self-motion; ENVI, perception of environmental-motion.

The results of the spontaneous perception under each of the four visual-vestibular interaction conditions are presented in Figure 1. The results show that the motion under four visual-vestibular interaction conditions varies among the subjects indicating inter-individual difference (Figure 1). A part of the subjects perceive self-motion, while another part—environmental-motion. That varies depending on the visual-vestibular condition. Under open eyes during rotation and after the stop condition illusory rotation (self and environmental) in the direction opposite to that of the chair rotation dominates (*p* < 0.001). There is no significant difference between perception of the self- and the environmental-motions. The self- and environmental-motion perception in the direction of the chair rotation is experienced in 6.2% (*SE* = 3.5%) of the trials. While under open eyes during rotation and after the stop condition the visual afferentation is integrated with the vestibular one, under closed eyes during rotation and after the stop condition the subjects had total visual deprivation. In the latter condition the perception of self-motion in the direction opposite to that of the chair rotation increased (*p* < 0.01). However, 16.7% (*SE* = 5.4%) of the subjects had a feeling of environmental-rotation, although they do not see it. Under this condition, the perception of self- or environmental-rotation in the direction of the chair rotation was rare–6.2% (*SE* = 3.5%) of the trials. In conditions of partial visual deprivation—closed eyes during rotation and open eyes after the stop condition and open eyes during rotation and closed eyes after the stop condition, different effects showed. In the former there is no significant difference between perceptions of the self- and environmental-motion perception in the direction opposite to that of chair rotation. In the latter, the perception of self-motion was higher and the perception of environmental-motion lower compared to both conditions with open eyes after the stop—closed and open during the rotation (*p* < 0.01). That is, in closed eyes during rotation and closed after the stop condition the results are closer to open eyes before and after the stop condition, while open eyes during the rotation and closed eyes after the stop condition shows results closer to closed eyes before and after the stop condition. Self- and environmental-motion perception in the direction of the chair rotation were rare experienced–6.2% (*SE* = 3.5%) of the trials.

Under the two conditions with closed eyes after the cessation of the rotation a third, unclear sensation appeared. Subjects described it as a spatial “disorientation.” By description it corresponds partly to “visually-induced dizziness” of the Barany Society classification (22). Disorientation dominates in open eyes during rotation and closed after the stop condition (*p* < 0.05).

In the spontaneous perception series, the gender effect was not significant.

**Figure 2:**
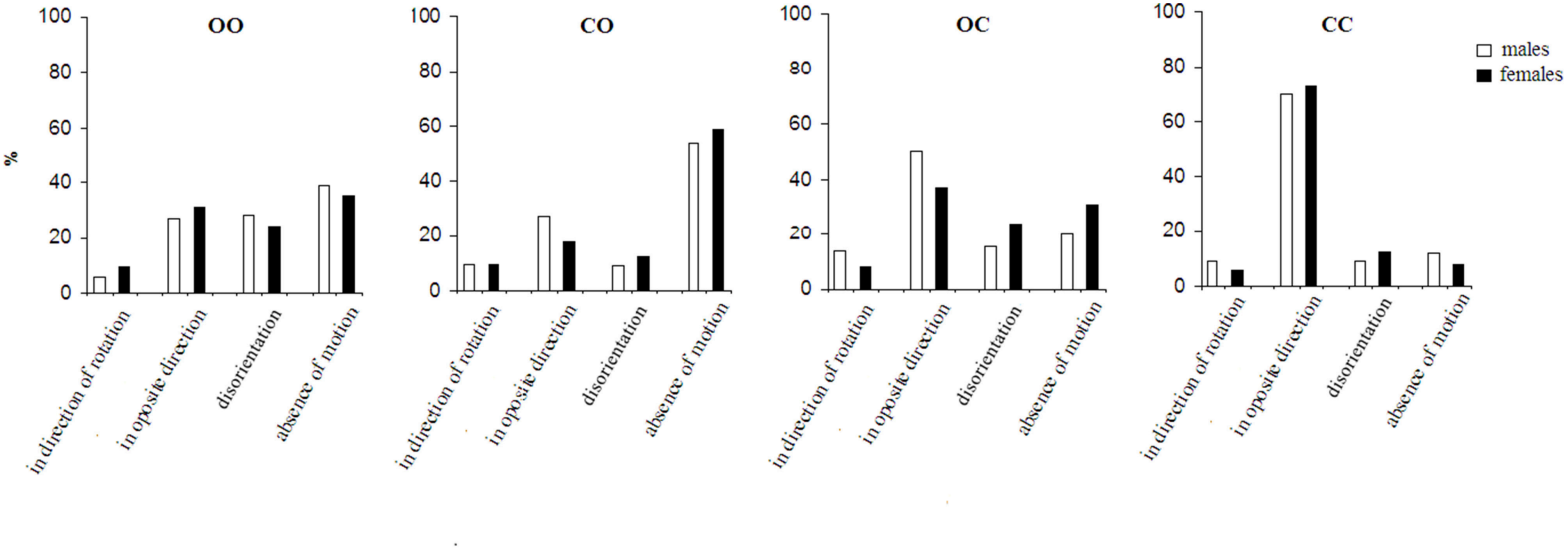
The perception of motion under conditions for the insinuation of self-motion. For the abbreviations see Figure 1.

**Figure 3:**
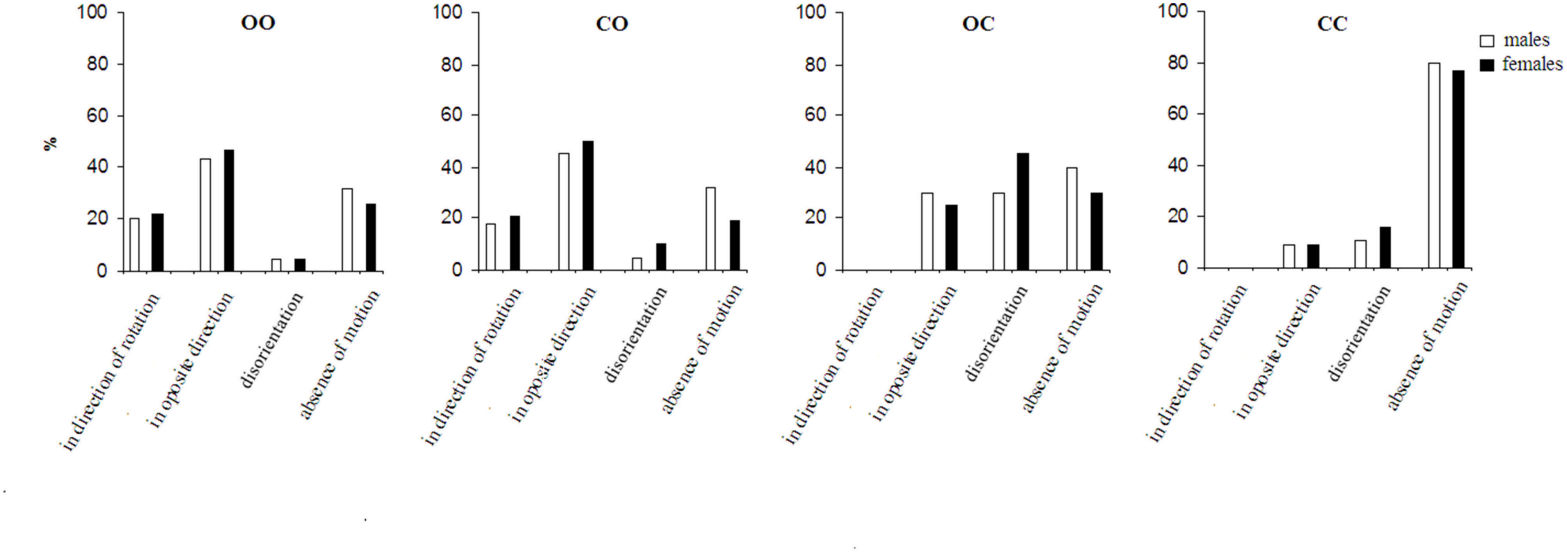
The perception of motion under conditions for the insinuation of environmental-motion. For the abbreviations see Figure 1.

The effect of the factor of insinuation on the perception of self-motion and environmental-motion is shown in Figures 2, 3. Generally, in the insinuation series the sensation referred to as disorientation becomes consistently more pronounced (*p* < 0.001) and the absence of motion as well (*p* < 0.001). Figure 2 presents the effect of the insinuation of self-motion on the motion perception. Under the first condition (open eyes before and after the stop) the perception of self-motion in a direction opposite to the direction of the chair rotation predominates over that in the direction of the chair rotation (*p* < 0.001). The experience of disorientation appears under this condition unlike in to the spontaneous series. Absence of motion consistently increased compared to the spontaneous series (*p* < 0.001) and it is dominating sensation in this condition. Under the condition of total visual deprivation the perception of self-motion in the direction opposite to that of the chair rotation increases nearly doubly compared to the first condition (*p* < 0.001). It strongly dominates all other sensations (*p* < 0.001). The disorientation sensation decreases almost doubly (*p* < 0.001), while the absence of motion sensation decreases nearly three times (*p* < 0.001). Compared to open eyes before and after the stop condition the insinuation of self-motion during partial visual deprivation, i.e., eyes closed during the body rotation and open after the stop or open during rotation and close after the sudden stop, shows different perceptual changes. In the former, the absence of the perception of motion dominates (*p* < 0.01), whereas in the latter, the perception of self-rotation in the direction opposite to that of the chair rotation dominates (*p* < 0.01). The disorientation sensation is present in both conditions but dominates under the condition with closed eyes after the sudden stop (*p* < 0.01).

Figure 3 presents the effect of the insinuation of environmental-motion on the motion perception.

Under the first condition, the perception of the environmental-motion in the direction opposite to that of the chair rotation significantly dominated all other sensations (*p* < 0.01) followed by absence of motion, motion in the direction of chair rotation and disorientation. Under the condition with total visual deprivation, although only in 8.3% (*SE* = 3.5%) of the trials there were reports of a perception of environmental-motion in a direction opposite to that of the chair rotation; however, nobody reported perception of environmental-motion in the direction of the chair rotation. Here, like the first condition there were reports of a disorientation sensation–14.6% (*SE* = 5.1%) of the trials. The absence of motion sensation strongly dominates under this fourth condition (*p* < 0.001) [79.2% (*SE* = 6.1%) of the trials]. With respect to the two conditions with partial visual deprivation—eyes closed during the rotation and opened after the stop and eyes open during the rotation and closed after the stop, the results of the former were close to the condition with open eyes before and after the stop. That is, perception of environmental-motion in a direction opposite to that of the chair rotation dominated (*p* < 0.01). Under the latter condition eyes open during the rotation and closed after the stop in a significant percent (*p* < 0.01) of trials–27% (*SE* = 4.4%), the subjects had a feeling of environmental-motion although they were with closed eyes. Under this condition the disorientation sensation slightly, insignificantly dominates, followed by an absence of any sensation of motion and environmental-motion in the direction opposite to chair rotation. Environmental-motion in the direction of the chair rotation was not experienced under this condition.

When both insinuation series are compared, it is seen that the insinuation effect differs significantly (*p* < 0.01) in the appearance of the insinuated motion perception (self- or environmental), as well as in the disorientation and the perception of an absence of motion. Under the most natural condition: with open eyes during and after the rotation, the insinuation modifies both perceptions in different ways. While in the spontaneous perception series there is no significant difference between the self- and environmental-motion perceptions under this condition, in the insinuation series the perception of environmental-motion dominates over self-motion (*p* < 0.05). This tendency of domination of environmental-motion is expressed more under the second condition—eyes closed during the rotation and opened after the stop (*p* < 0.01), while in the spontaneous series the effect is almost the same. Under the third condition—open eyes during the rotation and closed after the stop, there is an inversion of the effect: the self-motion perception dominates over the environmental-motion perception (*p* < 0.01). This perception dominates also, and is even more expressed, under the same condition, in the spontaneous series (*p* < 0.01). Under the last condition: total visual deprivation, the perception of environmental-motion consistently decreases, while the perception of self-motion correspondingly increases (*p* < 0.001). In the spontaneous series, respectively, the tendency is the same.

The disorientation perception significantly (*p* < 0.01) differs between the two insinuation series showing different tendencies across the four conditions. In the insinuation of self-motion it dominates under the first condition—open eyes before and after the stop (*p* < 0.01) but is more present in the insinuation of environmental-motion under the third condition—open eyes during the rotation and closed after the stop (*p* < 0.01). In the spontaneous perception series, the sensation is less expressed (*p* < 0.05).

With respect to the sensation of an absence of motion it also differs significantly between the two insinuation series. In the insinuation of self-motion it dominates under the second condition—closed eyes during the rotation and open after the stop over the other conditions (*p* < 0.01). However, in insinuation for environmental-motion it is most pronounced under the fourth condition—visual deprivation (*p* < 0.001).

The two insinuation series showed gender difference significant (*p* < 0.05) in both partial visual deprivation conditions (Figures 2, 3). It is pronounced for disorientation sensation—dominating in females, and absence of motion—dominating in males.

## Discussion

The present study found that after a sudden stop of constant velocity rotation humans with open eyes spontaneously perceive nearly equally either self- or environmental-motion, never both simultaneously. Presence or absence of visual cues prior or after the stop change the perception. Insinuation modifies the perception. There is a gender effect.

Self- and environmental-motion perceptions were investigated in different aspects using either vestibular—caloric or rotational, or visual stimuli [e.g., (11–14, 16–21, 23)]. When thresholds for motion were studied mostly sinusoidal visual and vestibular stimuli were used. Our previous studies (11, 13) showed how visual-vestibular interaction and the insinuation change the threshold for self-motion and object/visual scene motion perception during different frequencies sinusoidal rotation, to establish the frequency effect.

In the present study we used constant vestibular suprathreshold stimulus caused by sudden stop of constant velocity combined with different visual stimuli to establish the percent frequency of occurrence of perception for self- and environmental-motion.

This study shows that in healthy humans spontaneously under open eyes before and after the stop condition both types perceptions are presented almost equally. Our hypothesis is that the “egocentric” perception dominates in the brain of some humans while the perception of the external world (exocentric) dominates in others. We assume that it is possible for the mechanism to function at “a chance level” in some of the subjects (11). That is to say, their attention, at a particular moment, may be directed either toward the external world or toward themselves. The present study shows also that there are other factors which contribute to which type of perception is evoked. These are insinuation and the afferentation of visual cues integrated with vestibular afferentation.

It is interesting to note that even under total visual deprivation an environmental-motion perception can be created. One hypothetical explanation is Eigengrau/Eigenlicht phenomenon, more commonly referred to as visual noise. It is considered to be result of spontaneous discharge of the receptors in the retina which creates images (24). Supposedly such visual images contribute for motion perception. A second hypothesis, especially for open eyes during the rotation and closed after the stop condition, is that it is possible afterimage effect to facilitate the appearance of this perception. A recent study at threshold level indicates that afterimage lowers the threshold for self-motion perception (14). It might be at suprathreshold level this phenomenon to contribute for evoking perception of environmental-motion. We suppose that efference copy signal could probably contribute for evoking perception for the described environmental-motion. It provides the only extraretinal signal about eye position that is available without delay, and it is shown to be the most important extraretinal source of information for perceptual localization and motor activity. Efference copy accompanies all voluntary eye movements and some involuntary ones, including pursuits, saccades, and the fast phases of vestibular and optokinetic nystagmus (25). It could be admitted also that in the absence of visual afferentation from the external world an imaginary environmental image may exist in the brain, probably due to the short-term visuospatial residual memory for which the right parietal eye field and frontal eye fields play a key functional role as show several experiments (26–28). It is possible this perception to be generated by a combination of the proposed mechanisms.

The two types of partial visual deprivation used in this study showed different effects. The explanation could be that in the first case the visual signal is uninterruptedly moving with constant velocity along the retina while in the other this motion is caused by eye balls motion with decreasing velocity from the vestibuloocular reflex (VOR) which generates oculogyral illusion (29).

The effects of the insinuation on the perceptions of self- and environmental-motion are different depending on the condition which is in an agreement with our previous findings on threshold level in different experimental conditions of vestibular-visual interaction (11, 13). The evoked perception of environmental-motion dominates over the evoked perception of self-motion under the open eyes during rotation and after the stop condition, when the brain receives full visual afferentation in conjunction with those of the vestibular and the somatosensory ones. It dominates also, and is even more expressed, under the condition of reduced visual afferentation—closed eyes during the rotation and open after the stop, when the eyes are closed during rotation and opened after the sudden stop. Probably, the modulating influence of the insinuation has a stronger effect on the perception of environmental-motion than on that of self-motion. Probably to some extent this is constitutionally influenced as for instance the susceptibility to motion sickness (30) in which the vestibular and the visual systems are also involved (31, 32). In agreement with this hypothesis is that human behavioral genetic methods indicate individual behavioral difference with a genetic base (33).

It is interesting to note that the perceptions of self-motion and environmental-motion in the insinuated series do not dominate over the same perceptions in the spontaneous perception series. Even under the first three conditions they are slightly less perceived than that in the spontaneous perception series. This indicates that the insinuation influences mainly on the other two sensations: disorientation and absence of motion.

For the greater part of the trials the direction of the perceived two rotations is based on the functioning of vestibulospinal and vestibuloocular reflexes. However, in a small number of the trials there was a perception of rotation in the opposite direction. This probably is due to a perceptual signal intensity which, in this case, is close to the motion perception threshold but below to that for determining direction which, together with the existing noise in the system, causes an erroneous conclusion for motion direction (11, 19). In those who perceive “disorientation,” obviously the afferentation for motion perception is close but below the threshold for a definite motion. The signal is ineffective for the brain to define the motion.

Another interesting finding in this study is that nobody reported an appearance of both sensations simultaneously. The hypothetical explanation is that the motion perception mechanism is organized in such a way that probably only one perception can operate at a time especially when it is illusion. Once one illusory motion perception, evoked by suprathreshold motion stimulus, occupies the brain's perceptual mechanism, it does so completely until the sensory afferentation is changed. The phenomenon vection [described in number of studies, e.g., recent ones (34, 35)] supports this hypothesis. Once one is in vection it is so powerful illusory perception that he cannot stop this illusory motion perception. We hold that the phenomenon we describe belongs to the class of the Necker cube phenomenon, where perceptual interpretations tend to switch between two states. There exists a model that correspondingly describes the mechanism (36, 37). This class of models is called visual-vestibular conflict models. One may experienced a similar phenomenon also in the train illusion where one sits in a train and the one on the other track is moving, evoking a self-motion illusion that can switch back to feeling oneself stationary. There is a tendency that once one gets trapped into the illusion, it may be difficult to get out again. What is characteristic of such perceptions is that one can have only one or the other.

The results of the present study indicate that in human's brain perception there is significant gender effect in conditions of insinuation for motion. In agreement with this finding is that other human perceptive reactions also show a gender difference e.g., in neurosensory systems adaptation [to space in astronauts (38)]. The brain exhibits sex difference in responses to stress or other environmental cues (39), sensation and perception (40). Also in mental processes like mental rotation (41–43).

While in disease vertigo as a symptom is an indication for structural damage or alteration in the homeostasis, its biological meaning in healthy humans is unclear. The vestibular system and its interaction with the other systems in the brain are phylogenetically imperfectly created to function, thus evoking vertigo in healthy humans which in many situations is not useful, e.g., when appear oculogyral illusions (29), and in some cases even unpleasant.

Certainly, with the development of the transportation industry and especially astronautics and aviation, the importance of, and the necessity to understand, the nature of vertigo and its related sensations in healthy humans will increase; as will the need to manage them with new aproaches and devices due to the big problem of accidents caused by the human factor—spatial disorientation for motion, position, or attitude, especially in military aviation with loses, not only material, but also of human lives (9, 10). For instance the approach for creating artificial gravity by centrifuge for long-term space flights concerns the perception for motion (44).

The limitation of the present investigation is that the suprathreshold effect was studied for one suprathreshold stimulus only. In a next experiment it would be interesting to establish how increasing strength of suprathreshold stimuli affects the trend of the perception.

In conclusion, in healthy humans, after sudden cessation of rotation and with the subject's eyes open, the spontaneous illusory sensations of self- and environmental-motion are nearly equally presented; there is no simultaneous perception of illusory self- and environmental-motion; presence or absence of visual cues prior to the cessation of the rotation and immediately after the cessation of the rotation influence the perception of motion; there is an inter-individual difference in the motion perception; insinuation for either motion modifies the perception of motion; there is a gender effect.

## Author Contributions

The author confirms being the sole contributor of this work and has approved it for publication.

### Conflict of Interest Statement

The author declares that the research was conducted in the absence of any commercial or financial relationships that could be construed as a potential conflict of interest.
